# Thrombolysis in stroke patients: Comparability of point-of-care versus central laboratory international normalized ratio

**DOI:** 10.1371/journal.pone.0190867

**Published:** 2018-01-10

**Authors:** Ramona C. Dolscheid-Pommerich, Sarah Dolscheid, Lars Eichhorn, Birgit Stoffel-Wagner, Ingo Graeff

**Affiliations:** 1 Department of Clinical Chemistry and Clinical Pharmacology, University Hospital Bonn, Bonn, Germany; 2 Department of Rehabilitation and Special Education, University of Cologne, Köln, Germany; 3 Department of Anesthesiology and Intensive Care Medicine, University Hospital Bonn, Bonn, Germany; 4 Center Clinician Scientist, Emergency Department, University Hospital Bonn, Bonn, Germany; Maastricht University Medical Center, NETHERLANDS

## Abstract

**Background:**

In acute stroke patients, thrombolysis is one gold standard therapy option within the first four hours after the ischemic event. A contraindication for thrombolysis is an International Normalized Ratio (INR) value >1.7. Since *time is brain*, rapid and reliable INR results are fundamental. Aim was to compare INR values determined by central laboratory (CL) analyzer and Point-of-Care Testing(POCT)-device and to evaluate the quality of POCT performance in cases of potential therapeutic thrombolysis at a certified stroke unit.

**Methods:**

In 153 patients INR measurements using POCT-devices (HEMOCHRON Signature Elite®) were compared to INR measurements (BCS^®^XP) performed at the central laboratory. Outlier evaluation was performed regarding the critical thrombolysis cut-off.

**Results:**

Overall, we demonstrated a significant correlation (*r* = 0.809, *p*<0.0001) between both measurement methods. Mean value of the absolute difference between CL-INR and POCT-INR measurements was 0.23. In 95.4% of these cases, no differences regarding the critical cut-off (INR 1.7) were observed. POCT-INR values tended to be higher than the CL-INR values (*p* = 0.01). In 4.6% cases, a different value regarding thrombolysis cut-off was found. All patients were >75 years.

**Conclusions:**

POCT-INR measurements based on our POCT concept are suitable to determine INR values in critical stroke patients. Nevertheless, outlier evaluation is mandatory.

## Introduction

Stroke is one of the leading causes of death worldwide and a great global burden [1]. Although diagnosis and therapies have improved over time, thrombolysis with tissue plasminogen activators remains one gold standard therapy option within the first four hours after the ischemic event [2,3]. However, contraindications must be strictly considered when evaluating whether thrombolysis is an option. A laboratory analysis of, inter alia, International Normalized Ratio (INR) is mandatory in patients with acute stroke taking vitamin K-antagonists [4,5]. Guidelines refer to studies, which showed that an INR ≤1.7 revealed no elevated risk of hemorrhage in patients with acute ischemic stroke [6,7,8]. This important cut-off is crucial in the decision-making process regarding thrombolysis in ischemic stroke patients [5]. Reducing door-to-needle time is one of the aims of the guidelines for managing acute ischemic strokes given that studies demonstrated health benefits when reducing thrombolysis delays [4,6,9–10]. Point-of-Care testing (POCT) is expected to reach this aim since the main advantages of POCT are reduced transport times and sample preparation procedures. Reliable POCT-INR values are mandatory for thrombolysis not only at hospital stroke units, but also in recently developed mobile stroke units or pre-hospital settings [11]. Regarding POCT analysis of INR, it is important to consider that POCT devices for coagulation measurements deal with whole blood, whereas central laboratory analyzers use plasma. Therefore, known factors influencing POCT coagulation values, such as microaggregates, hematocrit variations and changes in plasma/cellular ratios, need to be considered by the clinician [12]. Proper training in POCT use and adherence to all internal and external quality control requirements is crucial not only for coagulation measurement, but all POCT measurements [13]. However, not all POCT devices fulfill the legally stipulated requirements. The guideline of the German Medical Association (Richtlinie der Bundesärztekammer, RiliBAEK) details all statutory requirements for quality control in POCT [14].

### Importance of the study

In stroke patients, thrombolysis is a therapy option which requires fast diagnosis. A rapid and reliable INR value is of fundamental importance to ensure immediate therapeutic consequences as a critical cut-off (INR ≤ 1.7) exists for thrombolysis. The effectiveness of POCT in reliably determining INR values to ensure immediate therapeutic consequences remains to be confirmed. Nevertheless, appropriate studies for all coagulation POCT devices are mandatory to evaluate this, while POCT is only an advantage when it is used correctly with reliable results.

### Aim of the study

Aim of the present study was to compare the values of the international normalized ratio (INR) determined by central laboratory analyzer (INR) BCS^®^XP and by coagulation POCT device HEMOCHRON Signature Elite® in patients presented at a neurological/stroke emergency department. This comparison was carried out to assess the quality of POCT performance at the University Hospital Bonn in cases of potential therapeutic thrombolysis and to evaluate whether in our setting, INR values determined by POCT yield reliable results that allow immediate therapeutic consequences in critically ill patients with indication for thrombolysis. Are POCT HEMOCHRON Signature Elite® INR results of reliable quality at our supra maximal care hospital to improve the management of acute ischemic strokes or do we have to provide an alternative central laboratory method for INR determination for our certified stroke unit?

## Materials and methods

### Setting

The University Hospital Bonn is a supra maximal care hospital with full research and teaching responsibilities; all medical disciplines are represented with 1250 beds; there are approx. 2600 POCT users. The hospital is certified according to DIN EN ISO 9001:2008 and the neurological emergency department/stroke unit fulfills all requirements for a certified stroke unit. The transport of blood samples via emergency blood delivery carriers follows standard operating procedures.

Blood sampling for POCT-INR and central laboratory INR measurements were carried out immediately after arrival of the patient. Absolute values of INR with a POCT device and via central laboratory analyzers were determined and specific cut-off values with diverging clinical relevance were analyzed.

### Ethics

The study is a single-center retrospective observational study. POCT as well as central laboratory samples were obtained as part of routine diagnosis. There was no additional collection of blood samples. Accordingly, our local ethic commission (Chairman K. Racké, MD, PhD, Professor, University Bonn, Germany) confirmed that the retrospective analysis of data obtained during routine treatment and diagnosis does not require consultation by the ethics commission according to §15 of the medical professional code. All collected clinical data evaluated in this study were fully anonymized before analysis. Therefore, according to prior agreement with the local ethics committee and the data protection officer appointed by the University Clinics Bonn, verbal or written informed consent was not obtained. As stipulated in article six of the German Data Protection Act (https://recht.nrw.de/lmi/owa/br_text_anzeigen?v_id=10000000000000000495#), the physician may use existing patient data for retrospective analyses without explicitly asking for the consent of patients. The study design is consistent with the Declaration of Helsinki.

### POCT concept at the University Hospital Bonn

We previously published our legally binding and law conforming POCT concept with a detailed description of responsibility levels, training conditions, quality control schemes and functions of the POCT commission [15]. At the University Hospital Bonn, three responsibility levels have been implemented: The medical director of the central laboratory and the POCT coordination are responsible for internal and external quality controls according to current legal regulations. Further, the POCT coordination is responsible for user training; user-to-user instruction is not permitted. Management of devices and back-up devices, user administration, hotline support, current device status, statistics of performance and checking for outliers also lie within the responsibility of the POCT coordination. At the University Hospital Bonn, trained users are responsible for patient samples, control sample measurements and refilling, emptying and ordering of consumables. Manufactures are responsible for repair and maintenance of the devices [15]. During the actual INR evaluation, our POCT concept remained unchanged.

### Quality controls

The guideline of the German Medical Association (RiliBÄK) stipulates that laboratory methods as well as POCT methods are subject to internal and external quality control. At our hospital, these quality controls are under the responsibility of the medical director of the central laboratory. According to RiliBÄK, the stipulated external quality control for INR requires participation in external quality assessment schemes. For both methods, the required conditions have been met regularly (Central laboratory- Coefficient of Variation (VC) and Standard Deviation (SD) were 2.3% and 0.48 respectively; INR target value 1.81 and POCT- VC and SD were 8.3% and 0.2 respectively; INR target value 2.5).

### Reference method central laboratory measurements

After centrifugation, reference method results for INR values at the central laboratory (CL-INR) were established in venous citrate plasma with Behring Coagulation System using the Dade^®^ Innovin^®^ assay (clotting detection) (BCS^®^XP) (Siemens Healthcare Diagnostics, Eschborn, Germany). For each new Dade^®^ Innovin^®^ lot a new INR calibration curve was generated according to the manufacturer’s instructions by using six calibrators (PT-Multi Calibrator^®^,Siemens Healthineers, Eschborn, Germany). Thrombocytes as platelet count and hematocrit values were obtained in whole blood EDTA samples with XN-9000™ (Sysmex, Norderstedt, Germany). Emergency mode analyses were performed in all samples immediately after arrival at the central laboratory. During the evaluation period, there was no change in analytical conditions, e.g. analyzers, methods and assays (prothrombin time reagents).

### POCT measurements

At the emergency department of the stroke unit, POCT-INR measurements were performed with the HEMOCHRON Signature Elite® Whole Blood microcoagulation system using the Prothrombin Time Assay (ITC®, San Diego, CA, USA) and the RecombiPlasTin^®^ reagent (IL, Lexington, MA) with an ISI of 1.0. Whole blood coagulation times were determined in vessels via LED detection of clots (mechanical endpoint clotting detection). The manufacturers instructions recommend that samples with a hematocrit <20% and >55% should be disregarded. All measurements were performed in single as part of routine diagnostic with the same POCT device. Measurements were performed by 20 trained users according to our POCT concept (see POCT concept at the University Hospital Bonn). During the evaluation period, there was no change in training, measurement or structural conditions.

### Patient collective/collected data

The patient collective included data from all patients (24 hours/ 7 days a week) between December 2015 and November 2016 from our certified stroke unit, in whom INR values were determined immediately after arrival in case of potential thrombolysis using POCT-INR and CL-INR measurements. In total 153 patients were included. 88 patients were taking anticoagulation therapies. 22 of these were taking vitamin K antagonists. All data were collected from the central laboratory information system (SWISSLAB II, Roche, Berlin Germany) according to the following analysis procedure:

1. Collation of the differences between POCT-INR and CL-INR measurements as absolute value of INR
2. Analysis of the critical thrombolysis INR cut-off > 1.7 –Values that differ with respect to the clinically relevant cut-off are subsequently called “above cut-off value”

Additionally, we investigated thrombocytes and hematocrit, which are known factors influencing INR measurement with POCT devices.

### Statistics

Data were statistically analyzed (Microsoft Excel, Version 2007; MedCalc® Version 11.0.0.0). *P* <0.05 was considered statistically significant. Age and absolute difference of INR values as well as thrombocytes and hematocrit are presented as mean value ± SD (standard deviation), minimum and maximum. INR values are described as mean value ± SD and male-to-female ratio. First, we performed correlation analyses by calculating Pearsons correlation coefficient in order to compare the INR measurement results. To provide sufficient results for a method comparison, we additionally summarized the data in a Bland-Altman plot, where the difference of POCT-INR and CL-INR (CL-INR—POCT-INR) was calculated for each patient and plotted against the mean value of both measurements ((CL-INR + POCT-INR)/2) [16]. We described the limits of agreement of the Bland-Altman plot (i.e. the interval within which 95% of differences between measurements by the two methods are expected to lie). Next, values exceeding the critical INR cut-off regarding the critical INR cut-off > 1.7 were analyzed. In addition, we compared both methods with respect to the (clinically) critical INR cut-off > 1.7. Potential differences between the two methods were analyzed by using cross tables and McNemar’s test. Values exceeding the critical INR cut-off > 1.7 were analyzed subsequently.

## Results

In our collective (N = 153 patients), male-to-female ratio was 95–58 (62.1% male and 37.9% female patients) with an average age of 72.9 years (range 15–97 years). Mean value of the absolute difference between CL-INR and POCT-INR measurements was 0.23 (range 0.0–3.0). Table 1 shows absolute differences between POCT-INR and CL-INR.

**Table 1:** Absolute difference between POCT-INR and CL-INR measurements.

Absolute Difference	Samples (%)
0.0	21 (13.73)
0.1	53 (34.64)
0.2	50 (32.70)
0.3	12 (7.84)
0.4	3 (1.96)
0.5	4 (2.61)
0.6	2 (1.31)
0.9	2 (1.31)
1.1	1 (0.65)
1.2	1 (0.65)
1.3	1 (0.65)
1.7	1 (0.65)
2.0	1 (0.65)
3.0	1 (0.65)

Table 1 shows the absolute differences between POCT-INR and CL-INR (absolute difference) (deviations in percentage distribution are due to rounding).

**Fig 1:**
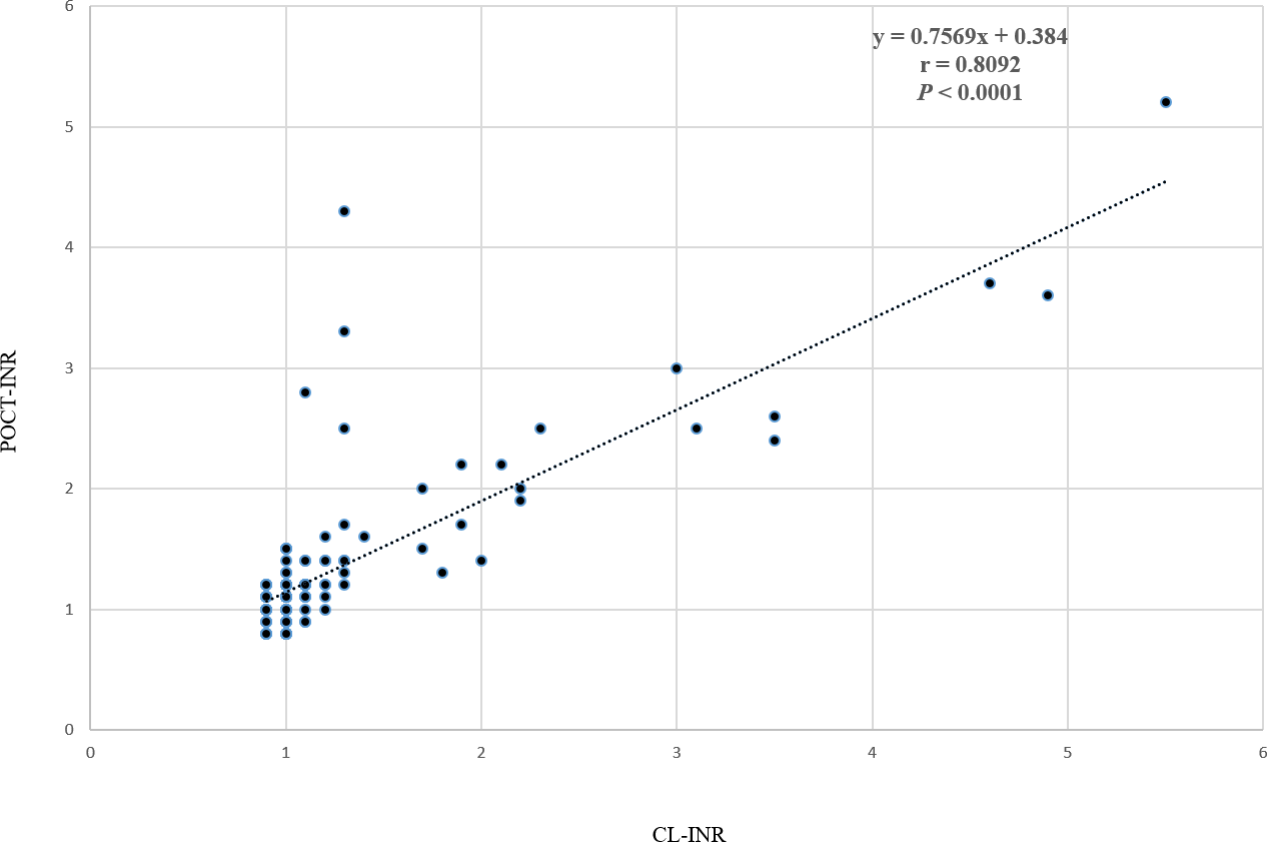
Correlation between CL-INR and POCT-INR. Fig 1 shows the correlation between the parameters CL-INR measured at the central laboratory with Behring Coagulation System (BCS XP) and POCT-INR measured at the neurology emergency department with HEMOCHRON Signature Elite® whole blood microcoagulation system.

**Fig 2:**
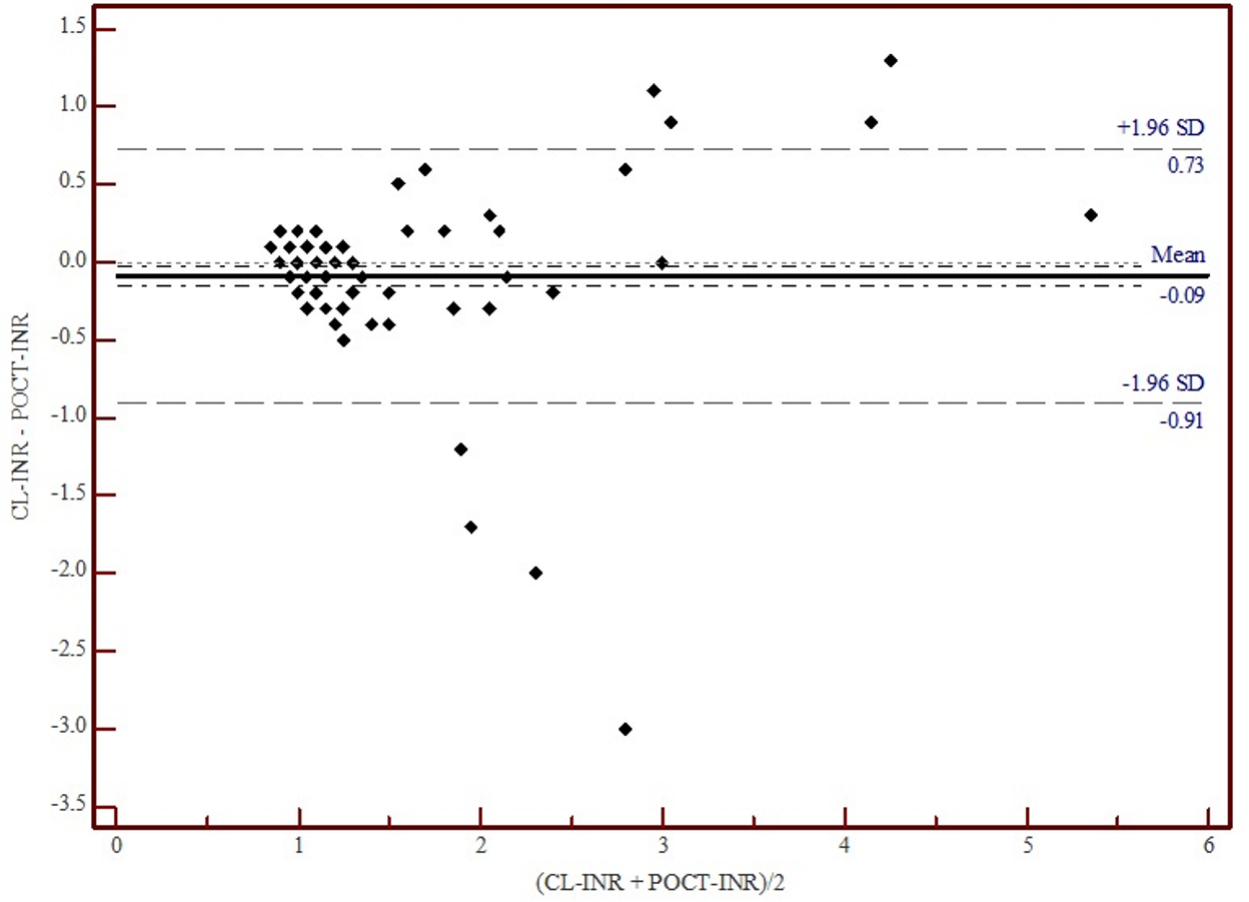
Bland-Altman plot showing the difference of the INR measurements. Fig 2 shows the difference of CL-INR and POCT-INR calculated for each patient and plotted against the mean value of both measurements (CL-INR + POCT-INR)/2). Main line shows the mean difference of the measurement values (0.09). The outer lines represent the limits of agreement within which 95% of differences between measurements by the two methods are expected to lie (-0.91 and +0.73).

For raw data, see S1 File. CL-INR mean value was 1.2 (SD 0.7), while POCT-INR mean value was 1.3 (SD 0.6). The correlation between CL-INR and POCT-INR was significant (*r* = 0.809, *p* < 0.0001) (Fig 1). On average, POCT-INR values tended to be higher than CL-INR values (*t* (153) *p* = 0.01), the mean difference of the measurement values was 0.09 (see Bland-Altman plot, Fig 2). The standard deviation of the differences was 0.36. Accordingly, the limits of agreement of the Bland-Altman plot range from -0.91 to + 0.73. Relevant values exceeding the critical INR cut-off were systematically analyzed below.

### Outliers

Table 2 shows the cross table for values exceeding the critical INR cut-off > 1.7.

**Table 2:** Cross table for the analyzed values exceeding the critical INR cut-off.

	POCT-INR In (%)	POCT-INR out (%)		Sensitivity	Specificity
CL-INR in (%)	134 (98.5)	5 (29.4)	139	98.53	70.6
CL-INR out (%)	2 (1.5)	12 (70.6)	24		
	136 (100)	17 (100)	153		

Table 2 shows the cross table with sensitivity and specificity for POCT measurement in cases of values exceeding the critical INR cut-off >1.7 for thrombolysis. “In” indicates that patients are classified for potential thrombolysis by the respective method (POCT, CL), whereas “out” indicates that patients fall outside of the potentially thrombolysis cut-off >1.7 according to the respective method.

McNemar’s test revealed no significant differences between the two methods regarding the thrombolysis cut-off > 1.7., *χ*^*2*^(1) = 0.57, *p* = .45, *ns*. Only in seven patients (4.58%) a clinically relevant and therefore important difference regarding the critical INR cut-off of > 1.7 for thrombolysis was observed. In these cases differences between both measurements were analyzed in detail. In this outlier collective, male to female ratio was 3 to 4, with a median age of 84 years (range 76–96 years). For CL-INR mean measurement was 1.5 (range 1.1–1.9) and for POCT-INR mean measurement was 2.6 (range 1.3–4.3). For thrombocytes mean measurement was 226 (range 160–298) and for hematocrit mean measurement was 40.7 (range 29–48). Detailed patients characteristics are shown in Table 3.

**Table 3:** Descriptive statistics of the analyzed values exceeding the critical INR cut-off.

Age	Sex	POCT-INR	CL-INR	Abs. Difference	Thrombocytes	Hematocrit
96	F	1.3	1.8	0.50	251	47
78	F	1.7	1.9	0.20	298	43
86	M	2.0	1.7	0.30	291	44
76	M	2.5	1.3	1.20	160	29
86	F	2.8	1.1	1.70	184	36
88	F	3.3	1.3	2.00	218	48
78	M	4.3	1.3	3.00	181	38

Table 3 shows data for INR, thrombocytes, abs. difference, hematocrit and patient characteristics with age and sex for patients with values exceeding the critical INR cut-off (abs = absolute).

## Discussion

In general, our results demonstrate reliable and valid POCT-INR values, as in 95.4% of cases, no differences regarding the critical thrombolysis cut-off (INR > 1.7) were observed. While on average POCT INR measurements tended to be higher than CL INR measurements, the absolute difference between the obtained INR values was very low (i.e. ≤ 0.2) in more than 80% of the samples. There was also a high correlation between both measurement methods. Finally, there were no significant differences between both measurement methods with respect to the critical thrombolysis cut-off (INR > 1.7), suggesting a good comparability of both POCT and CL INR measurements. In sum, our study revealed that the reported POCT INR measurements based on our POCT concept at UKB are suitable to determine INR values in critical stroke patients.

While previous studies have already reported excellent correlations between POCT- and CL- INR, only 32 patients were considered and a different POCT device was used [17]. Therefore, the validity is not transferable to a certified stroke unit as presented in our study. Studies with other POCT coagulation devices reported that INRs > 1.5 should be checked by a standard laboratory method as a high correlation was reported by previous studies [18,19]. In 39 patients with acute hemorrhage, INR determination with HEMOCHRON Signature Elite® revealed a lack of agreement in comparison to central laboratory results [20]. However, in addition to high overall correlations, it is also mandatory to analyze values exceeding the critical INR cut-off as we did.

Given that problems in INR-POCT determination may occur within the preanalytic phase as well as the analytic phase [21], we also investigated thrombocytes and hematocrit, which are known factors influencing INR measurement with POCT devices (cave at dehydration). The analysis of values exceeding the critical INR cut-off in our samples revealed that falsification might have happened in only one case. Our evaluation of values exceeding the critical INR cut-off with INR disagreement at the clinically important cut-off value of > 1.7 showed that all patients affected were >75 years of age. We therefore recommend being aware of those mismatches in older patients. While not all known influencing factors for POCT coagulation devices, such as microaggregation or hypothermia, can be detected, they must be considered when interpreting results [12]. Due to the different samples required (whole blood versus plasma), full agreement cannot be achieved between results from any POCT device and central laboratory results. Regarding Bland-Altman plot results in the therapeutic range for INR values (INR 2.0–3.5), the differences in variability between POCT and central laboratory measurements must be taken into account. This becomes obvious in quality controls which reveal that for POCT INR measurements, a maximal discrepancy of 32% from the target value is considered in line with legal requirements in Germany, whereas for CL INR measurements, the legal requirements stipulate a maximal deviation of 12%.

The problem regarding the two methods, that use a different thromboplastin reagent each, is widely discussed. Studies were carried out to assess these differences in INR values [22,23]. Riva et al. confirmed POCT devices as suitable when comparing with thrombin generation [24]. Baccouche et al. compared different reagent and instrument combinations and conclude that interpretation of data should be related to clinical intentions [25]. Nevertheless, only the combination of a valid POCT concept with intensive user training and troubleshooting can ensure reliable POCT results.

Analyses revealed that in other settings, the processing time of laboratory samples for INR measurements in stroke patients was too long [26]: The German Guideline “Acute therapy of ischemic stroke “and its 2015 addendum refers to Rizos et al., who state that POCT-INR measurements are sufficiently precise and reduce time intervals until thrombolysis [6,27]. The aim of our study was, inter alia, to provide sufficient data under actual conditions regarding legal updates (current RiliBAEK version) and analyze values exceeding the critical INR cut-off to help clinicians to decide whether it is useful to opt for INR values with standard laboratory procedure. The American guideline for early management of patients with acute ischemic stroke recommends INR determination in patients with oral anticoagulation with warfarin [4]. Understanding the reliability of POCT values is not only important, as we have shown, in our setting of a supra maximal care hospital, it also improves mobile stroke units, which are implemented in several states and countries. At mobile stroke units, no comparison with laboratory INR measurements is available. Thus, POCT values may not be reliable [28]. For mobile stroke units it is of fundamental importance to obtain reliable POCT-INR values and the organizational structures must be aware of ensuring appropriate responsibilities for external and internal quality controls.

Since *time is brain*, rapid diagnostic and therapeutic measures for acute stroke patients are recommended [4,9]. In case of non-reliable POCT-INR values, hospital settings must be optimized. At the University Hospital Bonn, a rapid blood transport system via couriers is available in case of emergency to reduce time delays.

### Limitations of the study

Preanalytical errors as well as different PT reagents can influence values in CL-INR and in POCT-INR. Economic observations, such as a cost benefit analysis, were not the focus of the present study. Also, we neither focused on anticoagulation therapies with vitamin K antagonists, nor on non-vitamin K antagonists. Other studies have been performed dealing with these issues [29,30]. In line with the established POCT concept at our hospital setting, only POCT device HEMOCHRON Signature Elite® and central laboratory BCS^®^XP were used.

## Conclusions

Overall, our study confirms that the presented POCT HEMOCHRON Signature Elite® INR values obtained using a POCT concept that is law conforming are reliable in a stroke unit situation regarding INR detection for thrombolysis in acute stroke patients. Nevertheless, evaluation of values exceeding the critical INR cut-off revealed discrepancies regarding the critical INR cut-off for thrombolysis in older patients (age > 75 years). Future studies should reproduce our findings with a broader population, not only in our setting, but also in stroke units elsewhere.

In line with our findings, we recommend performing evaluation studies of potentially discrepancies in values exceeding the critical INR cut-off in individual hospital settings to confirm the reliability of POCT-INR results. We further recommend implementing a central laboratory coagulation measurement back-up concept, where evaluation reveals discrepancies in values exceeding the critical INR cut-off to ensure reliable INR results. Our study indicates that age is a critical factor.

## Supporting information

S1 File Raw data. Raw data including POCT INR, Central laboratory INR and absolute difference.
(DOCX)
