# Supplementary material for: Thrombolysis in stroke patients: Comparability of point-of-care versus central laboratory international normalized ratio
Source: PLoS One. 2018 Jan 10;13(1):e0190867. doi: 10.1371/journal.pone.0190867 (PMC5761884; doi:10.1371/journal.pone.0190867)
Supplement: S1 File — Raw data including POCT INR, Central laboratory INR and absolute difference. (DOCX) [file pone.0190867.s001.docx]

|  | POCT-INR | CL-INR | Abs. difference for INR |
| --- | --- | --- | --- |
|  |  |  |  |
| 1 | 0.8 | 0.9 | 0.10 |
| 2 | 0.8 | 0.9 | 0.10 |
| 3 | 0.8 | 0.9 | 0.10 |
| 4 | 0.8 | 0.9 | 0.10 |
| 5 | 0.8 | 1.0 | 0.20 |
| 6 | 0.8 | 1.0 | 0.20 |
| 7 | 0.8 | 1.0 | 0.20 |
| 8 | 0.8 | 1.0 | 0.20 |
| 9 | 0.8 | 1.0 | 0.20 |
| 10 | 0.8 | 1.0 | 0.20 |
| 11 | 0.8 | 1.0 | 0.20 |
| 12 | 0.8 | 1.0 | 0.20 |
| 13 | 0.8 | 1.0 | 0.20 |
| 14 | 0.8 | 1.0 | 0.20 |
| 15 | 0.9 | 0.9 | 0.00 |
| 16 | 0.9 | 0.9 | 0.00 |
| 17 | 0.9 | 0.9 | 0.00 |
| 18 | 0.9 | 1.0 | 0.10 |
| 19 | 0.9 | 1.0 | 0.10 |
| 20 | 0.9 | 1.0 | 0.10 |
| 21 | 0.9 | 1.0 | 0.10 |
| 22 | 0.9 | 1.0 | 0.10 |
| 23 | 0.9 | 1.0 | 0.10 |
| 24 | 0.9 | 1.1 | 0.20 |
| 25 | 0.9 | 1.1 | 0.20 |
| 26 | 1.0 | 0.9 | 0.10 |
| 27 | 1.0 | 0.9 | 0.10 |
| 28 | 1.0 | 0.9 | 0.10 |
| 29 | 1.0 | 0.9 | 0.10 |
| 30 | 1.0 | 0.9 | 0.10 |
| 31 | 1.0 | 0.9 | 0.10 |
| 32 | 1.0 | 0.9 | 0.10 |
| 33 | 1.0 | 0.9 | 0.10 |
| 34 | 1.0 | 0.9 | 0.10 |
| 35 | 1.0 | 1.0 | 0.00 |
| 36 | 1.0 | 1.0 | 0.00 |
| 37 | 1.0 | 1.0 | 0.00 |
| 38 | 1.0 | 1.0 | 0.00 |
| 39 | 1.0 | 1.0 | 0.00 |
| 40 | 1.0 | 1.0 | 0.00 |
| 41 | 1.0 | 1.0 | 0.00 |
| 42 | 1.0 | 1.0 | 0.00 |
| 43 | 1.0 | 1.0 | 0.00 |
| 44 | 1.0 | 1.0 | 0.00 |
| 45 | 1.0 | 1.0 | 0.00 |
| 46 | 1.0 | 1.1 | 0.10 |
| 47 | 1.0 | 1.2 | 0.20 |
| 48 | 1.1 | 0.9 | 0.20 |
| 49 | 1.1 | 0.9 | 0.20 |
| 50 | 1.1 | 0.9 | 0.20 |
| 51 | 1.1 | 0.9 | 0.20 |
| 52 | 1.1 | 0.9 | 0.20 |
| 53 | 1.1 | 0.9 | 0.20 |
| 54 | 1.1 | 1.0 | 0.10 |
| 55 | 1.1 | 1.0 | 0.10 |
| 56 | 1.1 | 1.0 | 0.10 |
| 57 | 1.1 | 1.0 | 0.10 |
| 58 | 1.1 | 1.0 | 0.10 |
| 59 | 1.1 | 1.0 | 0.10 |
| 60 | 1.1 | 1.0 | 0.10 |
| 61 | 1.1 | 1.0 | 0.10 |
| 62 | 1.1 | 1.0 | 0.10 |
| 63 | 1.1 | 1.0 | 0.10 |
| 64 | 1.1 | 1.0 | 0.10 |
| 65 | 1.1 | 1.0 | 0.10 |
| 66 | 1.1 | 1.0 | 0.10 |
| 67 | 1.1 | 1.0 | 0.10 |
| 68 | 1.1 | 1.0 | 0.10 |
| 69 | 1.1 | 1.0 | 0.10 |
| 70 | 1.1 | 1.0 | 0.10 |
| 71 | 1.1 | 1.0 | 0.10 |
| 72 | 1.1 | 1.0 | 0.10 |
| 73 | 1.1 | 1.0 | 0.10 |
| 74 | 1.1 | 1.0 | 0.10 |
| 75 | 1.1 | 1.1 | 0.00 |
| 76 | 1.1 | 1.1 | 0.00 |
| 77 | 1.1 | 1.1 | 0.00 |
| 78 | 1.1 | 1.2 | 0.10 |
| 79 | 1.2 | 0.9 | 0.30 |
| 80 | 1.2 | 0.9 | 0.30 |
| 81 | 1.2 | 0.9 | 0.30 |
| 82 | 1.2 | 0.9 | 0.30 |
| 83 | 1.2 | 1.0 | 0.20 |
| 84 | 1.2 | 1.0 | 0.20 |
| 85 | 1.2 | 1.0 | 0.20 |
| 86 | 1.2 | 1.0 | 0.20 |
| 87 | 1.2 | 1.0 | 0.20 |
| 88 | 1.2 | 1.0 | 0.20 |
| 89 | 1.2 | 1.0 | 0.20 |
| 90 | 1.2 | 1.0 | 0.20 |
| 91 | 1.2 | 1.0 | 0.20 |
| 92 | 1.2 | 1.0 | 0.20 |
| 93 | 1.2 | 1.0 | 0.20 |
| 94 | 1.2 | 1.0 | 0.20 |
| 95 | 1.2 | 1.0 | 0.20 |
| 96 | 1.2 | 1.0 | 0.20 |
| 97 | 1.2 | 1.0 | 0.20 |
| 98 | 1.2 | 1.0 | 0.20 |
| 99 | 1.2 | 1.0 | 0.20 |
| 100 | 1.2 | 1.0 | 0.20 |
| 101 | 1.2 | 1.0 | 0.20 |
| 102 | 1.2 | 1.0 | 0.20 |
| 103 | 1.2 | 1.0 | 0.20 |
| 104 | 1.2 | 1.0 | 0.20 |
| 105 | 1.2 | 1.0 | 0.20 |
| 106 | 1.2 | 1.0 | 0.20 |
| 107 | 1.2 | 1.1 | 0.10 |
| 108 | 1.2 | 1.1 | 0.10 |
| 109 | 1.2 | 1.1 | 0.10 |
| 110 | 1.2 | 1.1 | 0.10 |
| 111 | 1.2 | 1.1 | 0.10 |
| 112 | 1.2 | 1.1 | 0.10 |
| 113 | 1.2 | 1.1 | 0.10 |
| 114 | 1.2 | 1.2 | 0.00 |
| 115 | 1.2 | 1.2 | 0.00 |
| 116 | 1.2 | 1.3 | 0.10 |
| 117 | 1.3 | 1.0 | 0.30 |
| 118 | 1.3 | 1.0 | 0.30 |
| 119 | 1.3 | 1.3 | 0.00 |
| 120 | 1.3 | 1.8 | 0.50 |
| 121 | 1.4 | 1.0 | 0.40 |
| 122 | 1.4 | 1.1 | 0.30 |
| 123 | 1.4 | 1.1 | 0.30 |
| 124 | 1.4 | 1.2 | 0.20 |
| 125 | 1.4 | 1.2 | 0.20 |
| 126 | 1.4 | 1.3 | 0.10 |
| 127 | 1.4 | 1.3 | 0.10 |
| 128 | 1.4 | 2.0 | 0.60 |
| 129 | 1.5 | 1.0 | 0.50 |
| 130 | 1.5 | 1.0 | 0.50 |
| 131 | 1.5 | 1.0 | 0.50 |
| 132 | 1.5 | 1.7 | 0.20 |
| 133 | 1.6 | 1.2 | 0.40 |
| 134 | 1.6 | 1.4 | 0.20 |
| 135 | 1.7 | 1.3 | 0.40 |
| 136 | 1.7 | 1.9 | 0.20 |
| 137 | 1.9 | 2.2 | 0.30 |
| 138 | 2.0 | 1.7 | 0.30 |
| 139 | 2.0 | 2.2 | 0.20 |
| 140 | 2.2 | 1.9 | 0.30 |
| 141 | 2.2 | 2.1 | 0.10 |
| 142 | 2.4 | 3.5 | 1.10 |
| 143 | 2.5 | 1.3 | 1.20 |
| 144 | 2.5 | 2.3 | 0.20 |
| 145 | 2.5 | 3.1 | 0.60 |
| 146 | 2.6 | 3.5 | 0.90 |
| 147 | 2.8 | 1.1 | 1.70 |
| 148 | 3.0 | 3.0 | 0.00 |
| 149 | 3.3 | 1.3 | 2.00 |
| 150 | 3.6 | 4.9 | 1.30 |
| 151 | 3.7 | 4.6 | 0.90 |
| 152 | 4.3 | 1.3 | 3.00 |
| 153 | 5.2 | 5.5 | 0.30 |
